# No increased in utero and peripartum HIV acquisition risk in HIV-exposed preterm infants

**DOI:** 10.4102/sajhivmed.v24i1.1509

**Published:** 2023-10-19

**Authors:** Gbolahan Ajibola, Charlotte Mdluli, Kara Bennett, Maureen Sakoi, Oganne Batlang, Joseph Makhema, Shahin Lockman, Roger Shapiro, Landon Myer, Kathleen Powis

**Affiliations:** 1 Botswana Harvard AIDS Research Institute, Gaborone, Botswana; 2 Bennett Statistical Consulting Inc, New York, United States of America; 3 Department of Infectious Diseases, Brigham and Womens Hospital, Boston, United States of America; 4 Department of Immunology and Infectious Diseases, Harvard T.H. Chan School of Public Health, Boston, United States of America; 5 Department of Public Health and Family Medicine, Faculty of Health Sciences, University of Cape Town, Cape Town, South Africa; 6 Department of Internal Medicine and Pediatrics, Massachusetts General Hospital, Boston, United States of America

**Keywords:** HIV acquisition risk, preterm neonates, vertical transmission, women living with HIV, antiretroviral treatment

## Abstract

**Background:**

Limited data exist on the differential risk of HIV acquisition between infants born preterm versus those born at term to women living with HIV (WLHIV). With a reported increase in preterm delivery among pregnant WLHIV, understanding the risk of vertical transmission of HIV in preterm infants can inform strategies to optimise the timing of diagnostic testing, antiretroviral prophylaxis, and infant feeding.

**Objectives:**

To describe the prevalence and timing of HIV acquisition, in utero versus perinatal, among infants with perinatal HIV exposure born prior to 37 weeks completed gestation age compared to those born at term in the Botswana-based Mpepu study and explore predictors of infant HIV acquisition.

**Method:**

Using data extracted from the Mpepu study, we describe the prevalence, timing and risk factors for HIV acquisition in infants born preterm versus those born at term. Fisher exact testing was used to test for differences in prevalence and timing of HIV and a multivariable logistic regression model was used to assess risk factors for infant HIV acquisition.

**Results:**

2866 infants born to WLHIV were included in this secondary analysis. 532 (19%) were born preterm. There was no observed difference in the prevalence of HIV acquisition among infants born preterm versus at term overall (0.8% vs 0.6%, *P* = 0.54), at birth (0.2% vs 0.3%, *P* = 1.00) or between 14 and 34 days post-delivery (0.6% vs 0.3%, *P* = 0.41). The absence of maternal antiretroviral use during pregnancy significantly predicted infant HIV acquisition, with the risk of HIV acquisition reduced by 96% among infants whose mothers were taking antiretroviral treatment (ART) during pregnancy (adjusted odds ratio: 0.003, confidence interval: 0.01–0.02, *P* < 0.001).

**Conclusion:**

There was no observed increase of in utero and peripartum HIV acquisition among infants born preterm following foetal exposure to HIV compared to those born at term.

**What this study adds:** Provides further insight into understanding the risk of HIV acquisition attributable to being born preterm. This knowledge has the potential to inform public policy relative to prophylactic strategies for preterm HIV-exposed infants, potentially contributing to a reduction in infant HIV acquisition.

## Introduction

Botswana provides a robust HIV treatment and prevention programme free of charge to its citizens and was one of the first countries in sub-Saharan Africa to offer comprehensive prevention of mother-to-child transmission of HIV (PMTCT) programming.^1,2^ Following updated World Health Organization (WHO) recommendations in 2016, Botswana scaled up its HIV treatment and prevention programming, initially expanding access to antiretroviral treatment (ART) for pregnant women living with HIV (WLHIV) to be lifelong, but ultimately offering lifelong ART to all persons with HIV, regardless of their disease stage at the time of diagnosis.^3^ These policy changes have been well implemented, with over 95% of pregnant WLHIV in Botswana having access to ART,^4^ resulting in a low vertical transmission rate of < 2%.^5^ This welcome achievement, however, means that an increasing number of infants are now exposed to antiretroviral drugs (ARVs) in utero, with ARVs potentially contributing to increased birth prior to 37 weeks gestational age, or preterm birth (PTB).^6,7,8,9,10,11,12,13,14,15,16^

Currently, PTB among HIV-exposed infants in Botswana is approximately 22% in comparison to 13% among HIV-unexposed infants.^17,18^ This figure is expected to rise as ART use in WLHIV increases, and an increasing number of WLHIV conceive while taking ART. In anticipation of this increase in PTB rates among pregnant WLHIV, it is imperative to understand if a differential risk of HIV acquisition exists among HIV-exposed infants born preterm in contrast to those born at term, as literature on this is currently sparse and findings from this study might help inform on birth testing practices for preterm infants born to WLHIV.

Furthermore, WHO and Botswana’s PMTCT guidelines^3,19^ currently recommend a triple ARV prophylaxis strategy in the first 4–6 weeks of life for HIV-exposed infants deemed to be at ‘high risk’ of HIV acquisition. In these guidelines, PTB is not recognised as a ‘high risk’ characteristic, in and of itself. Therefore, in Botswana, preterm HIV-exposed infants lacking any other risk factor receive a low-risk HIV prophylactic regimen of single-dose Nevirapine within 72 h of birth and twice-daily dosing of Zidovudine for the first 28–30 days of life.

We describe the prevalence and timing of HIV acquisition, in utero versus perinatal, among infants with perinatal HIV exposure born prior to 37 weeks completed gestation age compared to those born at term in the Botswana-based Mpepu study. We also explore predictors of infant HIV acquisition.

## Methods

This study is a secondary analysis of data collected from a prospective cohort of HIV-exposed children and their mothers in Botswana enrolled into the ‘Mpepu study’. The Mpepu study was a randomised controlled trial (RCT) evaluating the potential survival benefit of cotrimoxazole (CTX) prophylaxis among HIV-exposed but uninfected (HEU) children in Botswana.^20^ The Mpepu study recruited pregnant WLHIV from public antenatal clinics and maternity wards in Gaborone (a city), Molepolole (a village), and Lobatse (a town, included up to August 2012). Enrolment of the mother-child dyad could occur during pregnancy or as late as the infant’s 34th day of life. Study visits occurred at birth (or enrolment), 14–34 days (randomised visit to double-blinded assignment to CTX versus placebo), 2, 3, 6, 9, 12, 15 and 18 months of life, with randomisation occurring at 28–34 days of life from May 2011 to January 2013 and 14–34 days thereafter following modification of the protocol. Per the Botswana government’s PMTCT programming at the time, guidelines advocated that pregnant WLHIV receive three-drug ART during pregnancy and breastfeeding and recommended that all neonates with HIV exposure received single-dose Nevirapine (sdNVP) at birth plus Zidovudine (ZDV) for prophylaxis in the first 4 weeks of life. Infant HIV DNA polymerase chain reaction (PCR) testing was performed at the birth and randomisation study visit, per protocol. For infants with an initial positive HIV DNA PCR test, repeat confirmatory testing was performed. A positive confirmatory test was the criteria for infant HIV diagnosis. In utero transmission was deemed to have occurred if positive PCR results were obtained from samples tested at birth coupled with a positive confirmatory test and presumed peripartum HIV acquisition if the positive PCR result was obtained at randomisation (14–34 days of life) or later in life after an initial negative result at birth.

From the curated data set, we describe the prevalence, timing and risk factors for HIV acquisition in infants born preterm versus those born at term. Fisher exact testing was used to determine the prevalence and timing of infants seroconverting at birth or within 72 h post-delivery (for in utero estimates) and those testing positive at 14–34 days after an initial negative result at birth (for peripartum estimates), while the risk for HIV acquisition was determined using logistic regression. Preterm was defined as birth earlier than 37 weeks gestation.

### Ethical considerations

The Health Research Development Committee of the Botswana Ministry of Health and Wellness (Health policy, development, monitoring and evaluation [HPDME] 13/18/1 VI [29]) and the University of Cape Town (Health Research Ethics Committee [HREC] reference number 591/2020) Faculty of Health Sciences Human Research Ethics Committee provided regulatory overview and approval for this secondary analysis. Consent verification for the use of data in future studies occurred for all participants included in this analysis before using their data.

## Results

**FIGURE 1:**
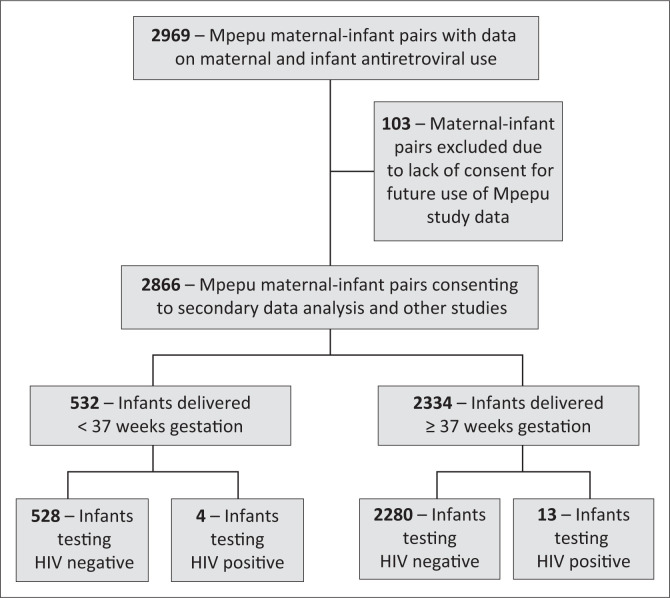
Study flowchart.

Data on 2866 of 2969 mother-infants pairs consenting for secondary use of data while participating in the Mpepu study were used for this analysis (Figure 1).

Of the 2866 included in this analysis, 2334 (81%) were born at ≥ 37 weeks’ gestation, while 532 (19%) were born prior to 37 weeks, completed gestational age. One thousand four hundred and sixty-one (51%) were female while 1405 (49%) were male. Median birth weight was 2.9 kg (range: 1 kg – 4.6 kg), with 493 (17.2%) weighing < 2.5 kg (low birth weight) at birth. Equal proportions of children were randomised to receive either CTX or placebo (1432 [50%] and 1434 [50%]), with randomisation occurring at a median age of 28 days (range: 14–34 days). Most infants were formula fed (79.1%), with only 20.6% breastfed from birth, 0.3% mixed fed. Average maternal age was 30 years (range: 18–47 years) and most mothers (82.5%) received a three-drug ART regimen either for prophylaxis or treatment during pregnancy, while 353 (12.3%) received just Zidovudine and 149 (5.2%) did not receive any treatment (Table 1).

**TABLE 1:** Demographics by infant HIV status.

Demographics	HIV status at birth					HIV status at 14–34 days				
	Negative (*n* = 2859)		Positive (*n* = 7)		*P*	Negative (*n* = 2849)		Positive (*n* = 10)		*P*
	*n*	%	*n*	%		*n*	%	*n*	%	
**Enrolment site**	-	-	-	-	0.697	-	-	-	-	0.418
Gaborone	1694	59.3	4	57.1	-	1689	59.3	5	50.0	-
Lobatse	214	7.5	0	0	-	214	7.5	0	0	-
Molepolole	951	33.3	3	42.9	-	946	33.2	5	50.0	-
**Infant sex**	-	-	-	-	0.279	-	-	-	-	0.754
Female	1459	51.0	2	28.6	-	1453	51.0	6	60.0	-
Male	1400	49.0	5	71.4	-	1396	49.0	4	40.0	-
**Birth weight category**	-	-	-	-	0.612	-	-	-	-	0.391
Low birth weight (< 2.5 kg)	493	17.2	0	0.0	-	490	17.2	3	30.0	-
Normal birth weight (≥ 2.5 kg)	2366	82.8	7	100	-	2359	82.8	7	70.0	-
**Infant prophylaxis**	-	-	-	-	-	-	-	-	-	0.795
ZDV	-	-	-	-	-	1553	54.5	7	70.0	-
NVP	-	-	-	-	-	1255	44.1	3	30.0	-
Unknown	-	-	-	-	-	41	1.4	41	1.4	-
**Gestational age at delivery category**	-	-	-	-	1.000	-		--	-	0.411
< 37 weeks	531	18.8	1	14.3	-	528	18.8	3	30.0	-
≥ 37 weeks	2287	81.2	6	85.7	-	2280	81.2	7	70.0	-
**Delivery mode**	-	-	-	-	0.561	-	-	-	-	0.437
Elective caesarean section	128	4.5	0	0	-	128	4.5	0	0	-
Emergent caesarean section	277	9.7	0	0	-	277	9.7	0	0	-
Vaginal delivery	2454	85.8	7	100	-	2444	85.8	10	100	-
**Duration of labour**	-	-	-	-	0.032	-	-	-		0.27
0 h – 6 h	754	31.5	1	20.0	-	751	31.5	3	33.3	-
6 h – 12 h	984	41.1	2	40.0	-	978	41.0	6	66.7	-
12 h – 24 h	612	25.5	1	20.0	-	612	25.6	0	0	-
> 24 h	15	1.9	1	20.0	-	45	1.9	0	0	-
**Gravidity**	-	-	-	-	0.495	-	-	-	-	0.807
Grand Multigravida	77	2.7	0	0	-	77	2.7	0	0	-
Multigravida	2386	83.5	5	71.4	-	2377	83.4	9	90.0	-
Primigravida	396	13.9	2	28.6	-	395	13.9	1	10.0	-
**Delivery regimen**	-	-	-	-	< 0.001	-	-	-	-	< 0.001
None	142	5.2	7	100	-	133	4.7	9	90.0	-
ZDV	353	12.3	0	0	-	353	12.4	0	0	-
HAART	2364	82.5	0	0	-	2363	82.9	1	10.0	-
**Maternal level of education**	-	-	-	-	0.905	-	-	-	-	0.178
None	54	1.9	0	0		53	1.9	1	10.0	-
Primary	409	14.3	1	14.3	-	409	14.4	0	0	-
Junior secondary	1622	56.7	5	71.4	-	1616	56.7	6	60.0	-
Senior secondary	555	19.4	1	14.3	-	552	19.4	3	30.0	-
Tertiary	219	7.7	0	0	-	219	7.7	0	0	-
**Household electricity**	-	-	-	-	0.261	-	-	-	-	1.000
No	1324	46.3	5	71.4		1319	46.3	5	50.0	-
Yes	1535	53.7	2	28.6	-	1530	53.7	5	50.0	-

s.d., standard deviation; ZDV, Zidovudine; HAART, highly active antiretroviral treatment; h, hours.

Overall, there were 17 infants who became HIV infected giving a mother-to-child transmission (MTCT) rate of 0.6%. There was no observed difference in the prevalence of HIV between infants born preterm versus those born at term overall (0.8% vs 0.6%, *P* = 0.54), at birth (0.2% vs 0.3%, *P* = 1.00) and at 14–34 days post-delivery (0.6% vs 0.3%, *P* = 0.41).

In univariable analysis, we observed a trend toward increased odds of infant HIV acquisition (in utero and peripartum) based on mother’s age at the time of delivery and ART use during pregnancy in WLHIV (Table 2). Odds of an infant being HIV positive by 34 days of life decreased as maternal age increased by a year and in infants born to WLHIV who were on ART for prophylaxis or treatment during the index pregnancy. Stratified by timing of HIV acquisition (in utero vs peripartum), infants born to WLHIV with a duration of labour lasting over 24 h had increased odds of acquiring HIV in utero while the odds of infant HIV acquisition in the peripartum period decreased as maternal age increased by a year and in infants born to WLHIV who were on ART for prophylaxis or treatment during the index pregnancy (Table 2).

**TABLE 2:** Predictors of positive HIV status.

Predictor	OR	95% CI	*P*	aOR	95% CI	*P*
**In utero HIV acquisition**						
Duration of labour (0 h – 24 h = Ref)	10.42	1.23 – 88.35	0.031	10.17	1.20 – 86.31	0.031
Gestational age category (< 37 weeks = Ref)	1.39	0.17 – 11.59	0.756	1.35	0.16 – 11.28	0.783
**Peripartum HIV acquisition**						
Maternal age	0.89	0.80 – 0.99	0.051	0.93	0.82 – 1.06	0.272
Delivery regimen (no ART = Ref)	0.006	0.001 – 0.05	< 0.001	0.006	0.001 – 0.05	< 0.001
Gestational age category (< 37 weeks = Ref)	0.54	0.14 – 2.10	0.374	1.55	0.38 – 6.42	0.542

OR, odds ratio; aOR, adjusted odds ratio; CI, confidence interval; Ref., referenced group.

In a multivariable analysis that included maternal age, delivery regimen, duration of labour (covariates with either a *P*-value ≤ 0.10 from the univariable analysis or significantly predictive of in utero or peripartum HIV acquisition) and adjusting for our a priori assumption that PTB would be associated with higher odds of infant HIV acquisition, maternal receipt of triple ART in pregnancy was significantly protective against infant HIV acquisition, decreasing the odds by approximately 96% (adjusted odds ration [aOR]: 0.003, confidence interval [CI]: 0.001–0.02, *P* < 0.001) (Table 2). Thus, the absence of antiretroviral regimens received by women during pregnancy was the only measured covariate in multivariable modelling that was independently predictive of HIV acquisition in children born to WLHIV in the first month of life.

## Discussion

We found no difference in the prevalence of HIV in HIV-exposed infants delivered preterm when compared to those delivered at term in the first month of life (0.8% vs 0.6%), and when stratified by timing of infection (at birth [0.2% vs 0.3%] and at 14–34 days post-delivery [0.6% vs 0.3%]) as postulated by the very few studies that have looked at this.^21,22,23,24^ Although we observed an increase in the prevalence of infant HIV acquisition, with 0.2% of infants testing positive at birth versus 0.6% testing positive at 30 days of life among infants preterm without an increase in the prevalence among infants born full term, this observed increase was non-significant. The absence of a significant difference in the overall prevalence of HIV acquisition between infants born preterm and those born full term or in the timing, in utero versus peripartum, in our cohort should be interpreted with caution as our sample size was small. Larger studies or pooling of data from similarly conducted studies are needed.

We found that non-use of antiretrovirals by women during pregnancy was the only measured covariate in our cohort that was independently predictive of HIV acquisition in children born to WLHIV, which re-affirms several other similar findings in literature.^25,26,27,28^ This also confirms that scaling up ART within programmes could help eliminate MTCT. Although lower than the reported 95% ART coverage from the Botswana national programme,^29,30^ the use of ART during pregnancy remained high, at 82.5%, in our cohort; this can be attributable to the fact that our study was conducted during the initial phase of the transitioning to triple ART use for PMTCT in Botswana. We reported a much lower MTCT rate of 0.6% in our cohort compared to the < 2% reported nationally^5^ confirming the effectiveness of triple ART in PMTCT as reported by several other studies globally and within the region.^31,32,33,34^ Similarly, we observed a high preterm delivery rate of 19% in WLHIV in a setting of high ART coverage consistent with reports from several other studies^15,16,35,36,37,38^ and corroborating findings suggesting in utero exposure to HIV and ART as possible drivers of the increased preterm delivery rates observed in WLHIV.

As mentioned above, the main limitation of this study is the very low MTCT rate observed; this could have reduced our power in detecting a true difference in risk of HIV acquisition between HIV-exposed infants delivered preterm versus those delivered at term. Also, our analysis was limited to 34 days post-delivery which could have led to underestimation of peripartum HIV acquisition rates in our cohort. This however is unlikely as participants in the Mpepu study were followed up for a period of 18 months and our review of the 18-month infant HIV status within the study did not report any new seroconversion occurring.

## Conclusion

In settings with low MTCT rates attributable to widespread use of triple ART regimen in pregnancy as in Botswana, there was no observed increase HIV acquisition risk overall, in utero and peripartum among HIV-exposed infants born preterm versus those born at term. However, findings from pooled data across several programmes would provide more power to detect any differential risk of HIV acquisition.
